# Supplementary material for: Causal Relationship between Aging and Anorexia Nervosa: A White-Matter-Microstructure-Mediated Mendelian Randomization Analysis
Source: Biomedicines. 2024 Aug 16;12(8):1874. doi: 10.3390/biomedicines12081874 (PMC11351342; doi:10.3390/biomedicines12081874)
Supplement: Supplementary file 1 [file biomedicines-12-01874-s001.zip › Supplementary Figure/Figure S4/Singe SNP plot of MR analysis for TL to ubm-b-1854.pdf]

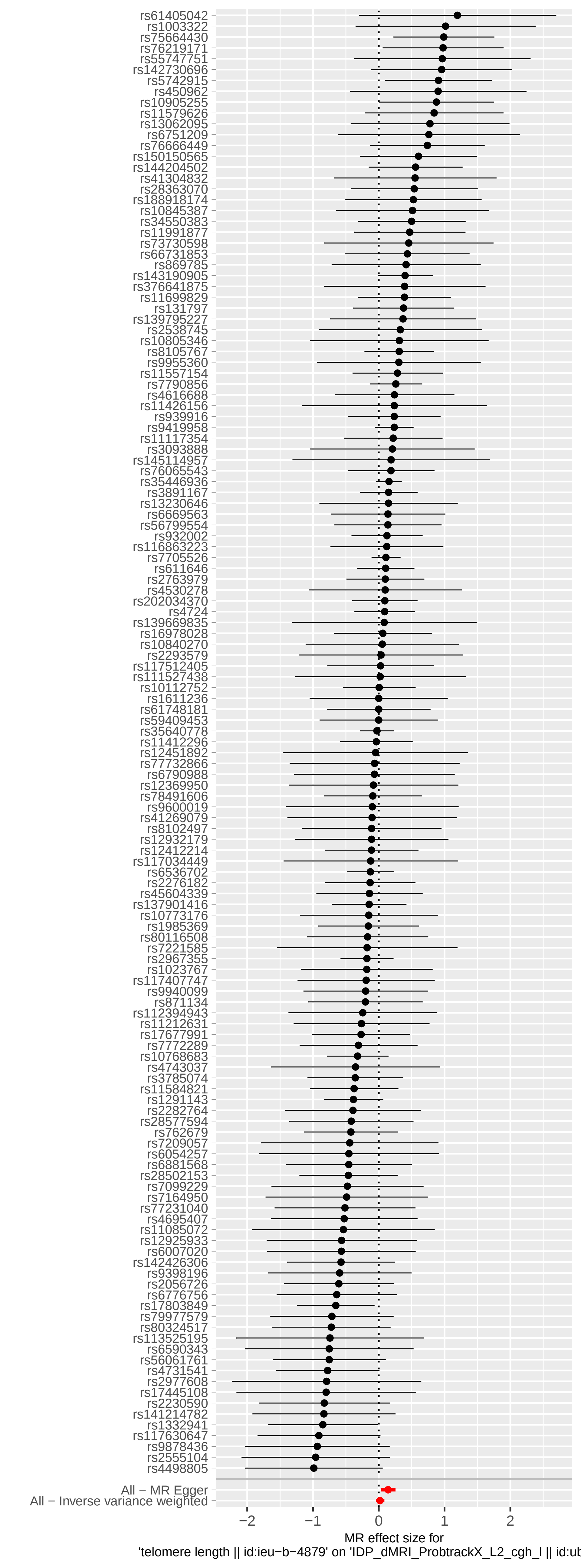
rs61405042
rs1003322
rs75664430
rs76219171
rs55747751
rs142730696
rs5742915
rs450962
rs10905255
rs11579626
rs13062095
rs6751209
rs76666449
rs150150565
rs144204502
rs41304832
rs28363070
rs188918174
rs10845387
rs34550383
rs11991877
rs73730598
rs66731853
rs869785
rs143190905
rs376641875
rs11699829
rs131797
rs139795227
rs2538745
rs10805346
rs8105767
rs9955360
rs11557154
rs7790856
rs4616688
rs11426156
rs939916
rs9419958
rs11117354
rs3093888
rs145114957
rs76065543
rs35446936
rs3891167
rs13230646
rs6669563
rs56799554
rs932002
rs116863223
rs7705526
rs611646
rs2763979
rs4530278
rs202034370
rs4724
rs139669835
rs16978028
rs10840270
rs2293579
rs117512405
rs111527438
rs10112752
rs1611236
rs61748181
rs59409453
rs35640778
rs11412296
rs12451892
rs77732866
rs6790988
rs12369950
rs78491606
rs9600019
rs41269079
rs8102497
rs12932179
rs12412214
rs117034449
rs6536702
rs2276182
rs45604339
rs137901416
rs10773176
rs1985369
rs80116508
rs7221585
rs2967355
rs1023767
rs117407747
rs9940099
rs871134
rs112394943
rs11212631
rs17677991
rs7772289
rs10768683
rs4743037
rs3785074
rs11584821
rs1291143
rs2282764
rs28577594
rs762679
rs7209057
rs6054257
rs6881568
rs28502153
rs7099229
rs7164950
rs77231040
rs4695407
rs11085072
rs12925933
rs6007020
rs142426306
rs9398196
rs2056726
rs6776756
rs17803849
rs79977579
rs80324517
rs113525195
rs6590343
rs56061761
rs4731541
rs2977608
rs17445108
rs2230590
rs141214782
rs1332941
rs117630647
rs9878436
rs2555104
rs4498805
All − MR Egger
All − Inverse variance weighted
−2
−1
0
1
2
MR effect size for
'telomere length || id:ieu−b−4879' on 'IDP_dMRI_ProbtrackX_L2_cgh_l || id:ub
